# Building capacity among health care librarians to teach evidence-based practice—an evaluation

**DOI:** 10.5195/jmla.2021.1126

**Published:** 2021-07-01

**Authors:** Abigail Sabey, Michele Biddle

**Affiliations:** 1 abby.sabey@uwe.ac.uk, National Institute of Health Research Applied Research Collaboration West and University of the West of England, Bristol, UK.; 2 michele.biddle@uwe.ac.uk, National Institute of Health Research Applied Research Collaboration West and University of the West of England, Bristol, UK.

**Keywords:** evidence-based practice, evaluation, capacity building, librarians, critical appraisal

## Abstract

**Objective:**

An innovative funding scheme for health care librarians to attend an intensive short course in teaching evidence-based practice was established in the West of England in 2016. This evaluation aims to understand the value of the scheme and the impact of the training opportunity for the librarians, establish an evidence base for continuing with the funding scheme, and inform the development of plans to build additional capacity among health care librarians to provide critical appraisal training.

**Methods:**

Seven librarians working in health care system settings were funded by the scheme between 2016 and 2018. Post-course feedback forms gathered initial views on course content and delivery, which informed the development of questions for the qualitative phase of the evaluation. All seven librarians participated in group discussions and individual interviews.

**Results:**

The course boosted confidence, provided valuable new skills, and positively impacted careers of the librarians through access to new opportunities. It inspired the development of new approaches to critical appraisal training. An important need was identified among the librarians for more education in teaching. Librarians funded by the scheme have successfully cascaded the training to their colleagues.

**Conclusion:**

This evaluation supports the continuation of the funding scheme to further build capacity among health care librarians to teach evidence-based practice. It suggests additional investment in this type of specialist training, as well as in education in teaching skills, would be beneficial for health care librarians. Evidence from this evaluation is informing new plans to support these professionals with the vital service they provide, which contributes to the evidence-based culture of their organizations and to patient outcomes.

## INTRODUCTION

The role of health care librarians in supporting evidence-based practice has long been acknowledged [1, 2]. In addition to expertise in searching and locating evidence, this specialist role, based in health care system settings, demands skills in critical appraisal, including knowledge of health research methods, and a degree of competence in explaining such ideas to others [3]. However, as librarianship training is based on a generalist approach, neither of these are formally taught [4]. This can leave a gap in skills and knowledge among health care librarians that presents a barrier to involvement in key parts of the role [5, 6], suggesting a need for more specialist training and support.

In 2014, Health Education England (HEE) set out an ambitious framework for modernizing the National Health Service (NHS) library and knowledge services to help achieve excellence in health care and health improvement [7]. A policy statement in 2016 renewed commitment to the development of NHS librarians to help mobilize research evidence in all levels of decision-making in health care to contribute to the drive for better health outcomes for patients [8]. As well as reorganizing services aligned with NHS priorities, this intended to improve training for librarians and enhance skills to meet the changing demands of the role. A national campaign followed that sought to promote the use of evidence in decision-making, based on the notion that a million decisions are made every day in the NHS which should be evidence based [9], raising demand for the expertise of health care librarians in critical appraisal.

It was within this context that the National Institute for Health Research Applied Research Collaboration West (NIHR ARC West, previously known as NIHR CLAHRC West) launched an innovative funding scheme for health care librarians in 2016. ARC West is one of fifteen organizations in England funded by the NIHR that brings together NHS, local authorities, and academic partners in the West of England to carry out research to improve health and health care in the local population and build capacity in research. A core strand of this work is dedicated to developing the skills of the health and social care workforce in research, evidence, and evaluation in its partner organizations [10]. As part of our strategy, we set out to build the capacity and capability of health care librarians to support evidence-based practice.

The aim of the ARC West health care librarians funding scheme is to support the development of teaching skills among local librarians, both to benefit library services and increase local capacity in critical appraisal training. This scheme funds local librarians to attend an intensive short course in Teaching Evidence-Based Medicine (EBM), run yearly at Oxford University. The scheme is advertised to all professionally qualified health care librarians working in the NHS in the West of England area (equivalent to twenty-three full-time staff) and offers funding to cover course fees (price in 2020 = £1350); usually two funded places are offered each year with scope to continue the scheme until 2024. Librarians apply to the scheme by an application form, providing information about their reasons for applying and how the learning would be applied to their future work. Successful applications to date have been those that demonstrated a strong interest in evidence-based practice and a commitment to deliver training in critical appraisal. The funding scheme also includes a commitment for each successful applicant to support joint events with ARC West to cascade the skills and knowledge acquired from the course to colleagues in local library services.

The Teaching EBM course is designed for health care professionals with existing knowledge of critical appraisal and experience in practicing evidence-based health care [11]. Each year the course attracts up to fifty participants from around the world to explore issues around teaching. Participants come from a mix of professions, with the largest group being clinicians. The most recent course content included separate sessions on how to teach searching methods, diagnostics, statistics for EBM, randomized controlled trials, and systematic reviews; developing a lesson plan; and EBM curriculum development and evaluation. Participants are facilitated in small groups to practice and develop their skills in teaching evidence-based practice effectively and with confidence. These lessons are important for health care librarians with roles in various aspects of the evidence-based practice teaching process [12]. However, other than those we have funded, only two attendees of the course since 2015 have been librarians.

This paper presents the findings of an evaluation of the ARC West funding scheme in its first three years, 2016 to 2018, to (1) understand the value of the scheme and the impact of the training opportunity for health care librarians, (2) establish an evidence base for continuing with the funding scheme, and (3) inform the development of plans to build additional capacity among health care librarians to provide critical appraisal training.

## METHODS

Ethical approval for this evaluation was granted by the Faculty of Health and Applied Sciences Ethics Committee at the University of the West of England, Bristol (Ref No: HAS.18.11.081).

### Data collection

In total, seven local librarians were funded under the scheme out of eleven applications in 2016–2018. Following the course, all were given evaluation forms devised by ARC West to gather their initial feedback on the course content and delivery (Appendix 1). These data were later used to help inform the questions for the qualitative phase of the evaluation. A short telephone interview was held with one of the seven librarians to explore which questions could be important to include in the final interview. Given the specific focus of the interview, it was not possible to pilot outside the group of funded librarians. The final interview topics included perceptions of their role in promoting evidence, previous training in evidence-based practice, value of the Teaching EBM course, specific examples of its impact, any personal benefits, views of the funding scheme opportunity, access to other continuing professional development (CPD), future plans to build on the learning, and any barriers to improving support for evidence use (Appendix 2).

Early in 2019, all seven funded librarians were invited to take part in a focus group for the evaluation. Due to workload pressures, four were unable to attend on the arranged date. Instead, two group discussions were held with three and two librarians respectively, and two librarians took part in individual interviews. Qualitative data collection took place between April and June 2019.

### Data analysis

A framework analysis was undertaken following the steps described by Gale et al. [13]. Data from the two group discussions and two interviews were transcribed verbatim by the lead researcher (AS); this contributed to the process of familiarization with the data. In addition, all recordings were listened to again during transcript checking. To facilitate the initial coding process, all data were entered into QSR NVivo 12. As advised by Gale et al., two researchers (AS and MB) independently coded the first transcript, and a simple coding structure was established. Through discussion, a second iteration of the framework was developed, which was then applied to all the transcripts to provide the basis for the charting stage. Charting sought to summarize the data in a matrix form whilst retaining the original meaning of the participants' words [11], and so this process included the selection of illustrative quotes for each category. This visual presentation of the data in a matrix supported the final stage of interpretation by facilitating the recognition of patterns in the data as well as anomalies and blank cells, helping to refine the final themes.

### Results

The analysis of the qualitative data revealed five themes as summarized in Table 1.

**Table 1.** Summary of themes

Themes	Subthemes
**The role of health care librarians in promoting evidence**	Librarians as evidence influencers and educators The changing role of librarians Critical appraisal Marketing
**Experiences of the Teaching EBM course**	Gaining a new perspective Feeling valued in the course Being inspired
**Personal benefits from attending the course**	Influence on career New skills Confidence
**Future plans**	Introducing new teaching Barriers to promoting evidence uptake
**Support of ARC West**	

### Theme 1: The role of health care librarians in promoting evidence

Librarians see themselves as being responsive to needs. Central to this is providing a timely, high-quality evidence service to all health professionals that helps to filter the large volumes of information and “make it a little more accessible” (P2). As this librarian explained, “I feel like [promoting evidence] that's our whole reason to exist as librarians” (P2). There were three areas within this theme which expand on the nature of the health care librarians' work.

#### 1.1 Librarians as evidence influencers and educators

In particular, librarians have a sense of responsibility for promoting the use of evidence, as well as educating staff. They talked of reminding clinicians about “the importance of evidence” (P2) and “questioning the evidence” (P1), and especially that “best evidence” is “the best evidence for your purposes” (P1), not what is determined by the evidence hierarchy. In this sense, librarians are advocates for evidence-based practice “and the core reason” (P6) behind it. This seems to be central not only to how they perceive their own role, but increasingly, how others perceive them. One librarian gave the example of being approached for help because “they've heard we know about research and stats” (P2) and can guide people through the steps of a systematic review, and another referred to helping other hospital departments meet their educational needs. Librarians are engaged in formal teaching, but this varies across different hospitals, with some being more limited in what they can offer.

#### 1.2 The changing role of librarians

##### 1.2.1 Critical appraisal

These discussions confirmed that the role of health care librarians has evolved in recent years, leading to greater demand for their involvement in supporting the critical appraisal of evidence and its subsequent uptake. This has reinforced the need to gain expertise in health care research and evidence. Compared to a few years ago, librarians have benefited from improved opportunities for external training, which they are encouraged to bring back and share with colleagues. Importantly, there is evidently huge support for CPD from the head of the local service: “he's brilliant, he's very forward thinking” (P7). This group of librarians were now (or had been) involved in some way in teaching critical appraisal, even those who were in their first job. For three librarians, there was a sense of being thrown in at the deep end, with two attending courses about teaching critical appraisal (in one case, this being the Teaching EBM course) in their first few weeks with no previous knowledge of this topic. This prompted one librarian to comment, “I do think teaching is something we should be learning” (P4), highlighting a significant gap in librarianship training (see also 3.2).

##### 1.2.2 Marketing

The changes to the role of health care librarians is driving a need for librarians to get involved in marketing. There were references in all the discussions to the importance of marketing library services: “You have to work hard in the NHS as a library service to build up your profile” (P6). There was also discussion around the struggle to get people to attend training, highlighting their drive to get more people using evidence and the importance of marketing as a skill needed by librarians: “a large part of it … is the marketing of services, so we've got all these amazing services but certain people don't know” (P7).

### Theme 2: Experiences of the Teaching EBM course

Overall, attending the course in Teaching EBM was a very positive experience for all the librarians: “I think it's invaluable really” (P1); “It was a great experience … we were very fortunate to be there” (P5), and prior to the ARC West scheme, there was “nothing on the scale [of the Oxford course]” (P6) available to the librarians. There were a number of specific elements that emerged as particularly positive.

#### 2.1 Gaining a new perspective

It was clearly beneficial for the librarians to be out of their usual context “in this world with experts” (P2) and learning from “brilliant teachers, brilliant figures in their fields” (P6). As well as being exposed to experts, there was value in learning alongside other professionals, and “talking to all sorts of people … was an amazing opportunity” (P4). This helped them to understand a different perspective on the value of critical appraisal and its application in the clinical context: “… having to see it from the position of the clinicians or nurses or whoever … was really important because we don't stop to think how anybody else is looking at it … why they're looking at it and what they're doing with it” (P3). Three librarians highlighted the international nature of the course participants in particular, “bringing ideas and showing different ways of teaching it” (P1) and the value of this kind of networking opportunity.

#### 2.2 Feeling valued in the course

Feeling valued as participants in the Teaching EBM course was important to these librarians, when normally, as one explained, “a librarian thing is to feel a bit of a fish out of water when you're teaching critical appraisal, to feel you're intruding on clinician territory” (P6). The experience was validating in the sense that it made them realize: “Yes, you have an area of expertise of equal value” (P6), and that they belonged alongside the clinical and academic participants in the course: “We didn't feel like 'another' in some ways … I think people were really pleased … to see librarians there and really valued our input and our role” (P1). The librarians also talked about sharing their knowledge with other participants and how “for them too it may have had value learning about our world” (P3).

#### 2.3 Being inspired

All the librarians in this study were inspired to change their teaching as a result of attending the Teaching EBM course or simply to learn more. Two were introducing critical appraisal teaching for the first time, which “wouldn't necessarily be something I would have done had I not done the course” (P7), and the other five made major improvements to their teaching offer: “We ended [up] going back and completely overhauling the way that we taught critical appraisal and our whole programme—we took a totally different approach after that” (P2), and “we threw out everything we used to do and redesigned everything” (P4). One particular example of being inspired was seeing how one of the course presenters constructed a session around a personal narrative: “we re-thought our teaching and tried to do something similar … a scenario we would follow through” (P6). Other more minor changes were triggered by the course too, such as being told “you don't have to teach statistics … get a researcher in to teach that, to teach it with you, share the work. I hadn't even thought of that” (P3). The course also prompted further learning, either in general: “It made me want to go away and find out more and explore more and teach myself more topics … it kind of inspired me” (P2), or about specific topics they were teaching, leading to other improvements to their sessions: “for instance with confidence intervals I've gone back and learnt some more and now made that more of the discussion” (P1).

### Theme 3: Personal benefits from attending the course

There were three areas where the librarians derived personal benefit from attending the Teaching EBM course.

#### 3.1 Influence on career

The course influenced the careers of most of the librarians in this study. Two were seconded to additional roles since the course, with one specifying that this was a higher-grade post and attributed this success, in part, to having done the Teaching EBM course: “I wouldn't have got into the position where I am now if I hadn't undertaken the course at Oxford … it's a step up” (P7). This individual was also, at the time of writing, considering taking the full postgraduate qualification in teaching evidence-based health care. The other librarian was offered a temporary transfer to a clinical department to help with data extraction for systematic reviews, broadening her experience outside her usual role. She explained how the course had been an eye opener, which made her realize: “health research is really interesting … we don't normally go so far into seeing all this research and all that's going on … that certainly contributed to this sort of interest that I'm developing” (P2). Two other librarians initiated research activities since the course, one following a conversation with a fellow participant about perceptions of clinical librarians: “I wouldn't have ever done that if I hadn't had that conversation on the course” (P3). The other had submitted their first abstract to a conference, which was accepted for a presentation: “I learnt a lot from it and I would never have done it if I hadn't been on the Oxford course … it was a huge thing for me” (P4). Finally, one librarian moved into a new job with a specific teaching and learning focus, referring to the course as life changing and tentatively making a connection between the course and the change of job: “I think the course empowered me to think about it [teaching] more … I was in a different mindset after that course” (P6).

#### 3.2 New skills

The course equipped all the librarians with valuable new tools for teaching, such as new frameworks for critical appraisal and helpful tips like “how to deliver this idea in a bite-sized kind of thing … you can explain something in five minutes” (P1) and “explaining the p value … in a different way” (P5). Constructive feedback on microteach sessions during the course similarly boosted teaching skills, such as being advised to “come in with something that will astound them … that really changed how I present … people respond differently when you engage with them … I try to be a little bit more creative” (P1). In addition, the course helped with the practice of teaching itself, such as learning about teaching plans that was described as “a lightbulb moment” (P4), as well as the realization that “you don't need to know everything, you just need to know how to control the group and facilitate” (P4). One librarian in their first job since graduating commented, “just to sit down to learn some stuff about critical appraisal and how to teach it … teaching is something I have never done previously so that was a really big element of it” (P7). This discussion again suggested a gap in training for health care librarians around teaching skills: “I do think teaching is something we should be learning and doesn't seem freely available or visible” (P4).

#### 3.3 Confidence

Boosting the confidence of librarians and their legitimacy as teachers was a clear outcome of the Teaching EBM course: “I really think the biggest thing for me was confidence because although I thought I knew some of the things it gave a fresh look on everything, yes, definitely the big thing for me was confidence” (P4). The opportunity to present in front of colleagues as part of the course was for many “way out of my comfort zone, it's not something I'd ever done before or even thought about doing … I felt a real sense of achievement” (P7). This in particular boosted the feeling of being valid teachers in this context: “it makes you feel you can do this, and this is all amongst all the other doctors and researchers and whoever else is there … and yeah, this is completely valid, we can do this, we can teach it” (P2), and “the course gave us that shot in the arm that we are really well placed to deliver on this” (P6). Just one participant felt differently, pointing out that the course also highlighted some gaps: “it reaffirmed some knowledge … but you do realise everything else you don't know” (P5).

### Theme 4: Future plans

As well as being a positive experience and having personal benefits for the librarians, the course in Teaching EBM impacted the future plans of these librarians.

#### 4.1 Introducing new teaching

There was a real enthusiasm for teaching among this group of librarians, and the course fueled ideas for new areas of teaching such as higher-level statistics and qualitative appraisal. One team was considering a more tailored approach to sessions: “We're thinking you need tiers, like different levels of presentation depending on the group of people you're delivering it to” (P4). There were plans in one case to reach out to new audiences in the hospital: “our commissioning team … the head of the team was very on board with the idea so it's just timing” (P4).

#### 4.2 Barriers to promoting evidence uptake

The librarians in this study recognized the challenges that came with their plans and ambitions for widening the teaching of evidence in their organizations. They perceived staff time to be the predominant barrier to engaging more staff in learning about and using evidence: “they just don't have time to come away from the clinical area any more” (P4). Most of them also felt their own lack of time was a barrier to how much teaching and support can be offered: “We have effectively 4000 people each, we can't look after 4000 people, not feasible” (P3), which understandably is a concern for the librarians: “I feel like we're spread a bit thin” (P1). This was linked to the importance of making sure “health librarians are trained to the best standard” (P3) as a way to equip them to cope with demand. The issue of clinicians' lack of access to computers on wards for point of care tools, such as DynaMed, was also mentioned as an onward barrier.

### Theme 5: Support of ARC West

Funding for the Teaching EBM course would have been a struggle without the ARC West scheme: “Financially it was completely outside of what we would have been able to do” (P2), and it was a significant opportunity for those health care librarians who were successful in securing a place: “not everyone has the chance to do the Oxford course … that was their main opportunity, their main event of the year” (P2). Participating in the wider ARC West training opportunities was also recognized as contributing to librarian training alongside local CPD or higher education and was helping to meet the needs of this group of professionals: “It's so difficult to get appropriate training … we are swimming uphill” (P3).

## DISCUSSION

This evaluation identifies a range of positive impacts from the ARC West funding scheme for health care librarians, at both personal and organizational levels, and how this is helping to grow capacity in teaching evidence-based practice. It also provides insight into the drive and commitment of these professionals to improve their services.

In addition to being inspiring, the opportunity to attend the Teaching EBM course clearly provided an important endorsement of the librarians' legitimacy as teachers of evidence-based practice and had a valuable impact on their confidence and willingness to get involved in teaching. Previous studies have similarly found that health care librarians lack confidence in teaching critical appraisal [2] or in their research knowledge [14] and in some cases may experience impostor syndrome in relation to their medical knowledge [15]. This evaluation reinforces the importance of the kind of specialist training enabled by the funding scheme in refreshing and boosting knowledge, skills, and confidence for the job of health care librarians, from which to build capability in teaching evidence-based practice. Beyond the ARC West context, this is especially important as demand for critical appraisal expertise is nurtured by policy initiatives. Given how few librarians other than those funded by our scheme attended the Teaching EBM course, consideration should be given to how wider access to such training for health care librarians could be enabled.

An unexpected benefit of the Teaching EBM course for some was its impact on careers, with two staff obtaining short-term transfers to promoted roles. For one, it gave them the chance to be more fully involved as part of a systematic review team, which is a less common but emerging area for librarians' involvement [16, 17], and for another, the experience prompted them to apply for a new role which they successfully secured. This underlines the importance of this kind of professional development in expanding such opportunities for library staff, especially those in highly pressured roles.

A further benefit was also the experience of learning with clinicians and academic staff, which helped the librarians to better understand the perspective of those using their services. The value of interprofessional training in helping health care staff from different professional groups work together effectively is well documented [18], specifically in the context of learning about evidence-based practice [19, 20]. A recent study highlights that health sciences librarians in particular are positive about such opportunities [21]. This may be useful to consider for future training activities for librarians at ARC West and elsewhere.

The funding scheme also delivered change at an organizational level. Attending the course brought fresh creativity and ambition, inspiring the development of new critical appraisal training and major improvements to existing teaching offered by librarians. The course generated ideas and plans for other additional training, such as offering different levels of critical appraisal courses and more tailored learning. As well as increasing local capacity for such training, these enhancements to critical appraisal training for clinical staff have potential to impact health care on a much wider scale through the greater use and application of evidence to patient care [22].

There are a number of implications from this evaluation for the future of librarians' training. First, a consistent theme was the lack of training in teaching itself, despite this being a central part of the health care librarian's role, reflected in the professional competencies of library associations in the United Kingdom, United States, and elsewhere [23, 24, 25]. A recent scoping review of the educational needs of specialist health care librarians working within clinical teams identified education as one of their main needs, with secondary needs including abilities to create information literacy educational programs, to provide the necessary education to clinical staff, and to teach information seeking skills [26]. A similar need is reflected in a recent survey of health care librarians in England that showed teaching and training skills featured in the top ten CPD requests [27]. Given the very high ratio of qualified librarians to NHS staff (1:1730) [28], which places huge pressure on individuals, this seems an urgent area to address. Teaching librarians how to teach would help support these professionals in a crucial part of their work and help them meet demand for their services. Similar calls for further training in teaching for health care librarians have been made by those working in such roles in the United States and Canada [29, 30, 31]. There was also the issue of marketing library services, a key skill for which librarians are not trained but could be usefully added to a CPD portfolio. Again, this featured highly as a CPD request in the recent survey of librarians in England [27].

There have been onward impacts from the scheme which further support its continuation. Five of the seven librarians in this evaluation successfully codelivered training for their colleagues, helping to cascade the learning from the Teaching EBM course. The first workshop in 2017 was attended by twelve local health care librarians, where two of our funded librarians contributed to sessions. This event focused on sharing tips from the Teaching EBM course as well as a wider discussion about the changing culture of evidence in health care and teaching other sectors of the health care workforce. This event was highly rated by participants (average 3.72/4, with 4 being excellent) with comments showing the value of sharing good practice and boosting skills in areas such as statistics. A second workshop was held in 2018, attracting a larger group (eighteen librarians) from beyond our local area, including some academic librarians who support health care students. This was codelivered with three of our funded librarians and focused on sharing best practice in teaching critical appraisal and was the first educational offering of its kind in the area covered by the scheme. Again, this event generated very positive feedback scores (average 3.71/4) with comments highlighting the value of being given new ideas and strategies for teaching. Thirteen participants indicated their commitment to build on the training to develop new or refresh existing critical appraisal teaching demonstrating the impact of the event.

These two events demonstrate the wider return on investment from this scheme. As a result of this evaluation, we are developing further plans for codelivered training at ARC West to continue building capacity in local library services for critical appraisal training. Moving to a sustainable model of training, the current plans are to support the librarians funded by the ARC West scheme to run regular bite-sized sessions on critical appraisal for their colleagues, expanding the cascade of learning, and growing more capacity for appraisal training. Through this collaboration, we aim to support this professional group in keeping their critical appraisal and training skills practiced and refreshed and to maintain their confidence. This in turn would enable them to offer more training sessions for their clinical communities.

A limitation of this study that affects its generalizability should be acknowledged. The focus on a discrete group of local health care librarians who were selected for the ARC West funding scheme necessarily restricted the sample size, and formal data saturation was not applicable. It is possible that we may have revealed different experiences with a larger group of librarians. However, it was noted during analysis that there was strong similarity among the views expressed by this group, and the discussion highlights key findings that are consistent with the wider literature, enhancing the plausibility of this evaluation. Overall, this study demonstrates the value of even small-scale evaluations of innovative schemes such as this one in establishing an evidence base for investment.

In conclusion, this positive evaluation of the ARC West funding scheme to build capacity among health care librarians to teach evidence-based practice supports its continuation and, furthermore, suggests that additional investment in this type of training for health care librarians would be beneficial. The scheme may be a useful exemplar for other organizations. The scheme and its evaluation helped inform new plans for supporting this group of professionals with the vital service they provide to their clinical colleagues, which contributes to the evidence-based culture of their organizations and to patient outcomes.

## Data Availability

The coding matrix from the framework analysis is available in the University of the West of England Research Data Repository at http://researchdata.uwe.ac.uk/616.

## SUPPLEMENTAL FILES

**Appendix 1:** ARC West post-course feedback form template

**Appendix 2:** Interview schedule
