# Supplementary material for: Building capacity among health care librarians to teach evidence-based practice—an evaluation
Source: J Med Libr Assoc. 2021 Jul 1;109(3):432–40. doi: 10.5195/jmla.2021.1126 (PMC8485954; doi:10.5195/jmla.2021.1126)
Supplement: Supplementary file 1 — Appendix 1: ARC West post-course feedback form template [file jmla-109-3-432-s01.docx]

##

## Overall

Please describe the area/s of the event that you found most helpful *(please continue overleaf if needed)*

Please describe the area/s of the event that you found least helpful and/or areas that could have been developed further *(please continue overleaf if needed)*

**Please circle the score that most closely represents your views on these aspects of the workshop:**

**Poor Fair Good Excellent**

| **Overall rating for the event** | **1** | **2** | **3** | **4** |
| --- | --- | --- | --- | --- |

**Sessions Poor Fair Good Excellent**

| **Content of Sessions** | **1** | **2** | **3** | **4** |
| --- | --- | --- | --- | --- |
|  | | | | |
| **Delivery of Sessions** | **1** | **2** | **3** | **4** |
|  | | | | |

**General Poor Fair Good Excellent**

| Hand outs | **1** | **2** | **3** | **4** |
| --- | --- | --- | --- | --- |
| Room | **1** | **2** | **3** | **4** |
| Admin/information supplied pre-event | **1** | **2** | **3** | **4** |

| **One action I’m going to take as a result of the course:** |
| --- |

**PTO**

**Would you recommend this course to your colleagues?**

**Do you have any other comments?**

**Thank you for giving your feedback - Please return by email to:**

***Workshop: Teaching Evidence Based Medicine*** | ***Date:***

***Venue/provider:*** Centre for Evidence-Based Medicine, Oxford
