# Supplementary material for: Building capacity among health care librarians to teach evidence-based practice—an evaluation
Source: J Med Libr Assoc. 2021 Jul 1;109(3):432–40. doi: 10.5195/jmla.2021.1126 (PMC8485954; doi:10.5195/jmla.2021.1126)
Supplement: Supplementary file 2 — Appendix 2: Interview schedule [file jmla-109-3-432-s02.docx]

*Opening:*

How would you describe your role in promoting the use of evidence?

What training have you had in the past in critical appraisal and/or teaching critical appraisal?

What CPD are you able to access as a librarian?

*Main topics:*

What did you find most valuable about the Oxford course? And least valuable?

How do you feel the training benefitted the service you provide in the library? Do you have any examples of something you have initiated or done differently at work as a result of the course?

Do you feel the Oxford course benefitted you personally? If so, in what ways?

What are your views of the ARC West funding scheme? (prompts: do you think there is value in ARC West continuing to provide the funding for the Oxford course? Would you have been able to secure funding for the course without the scheme?)

*(If applicable)*What do you feel you gained from working with ARC West to develop a new workshop for your colleagues?

Can you tell me about any plans you have to build on these experiences further in your library service?

Have you encountered any barriers to getting new initiatives to support evidence uptake, off the ground? Is there anything else that would help librarians support the uptake of evidence in practice?
